# Impact of environmental tax on green development: A nonlinear dynamical system analysis

**DOI:** 10.1371/journal.pone.0221264

**Published:** 2019-09-04

**Authors:** Xinghua Fan, Xuxia Li, Jiuli Yin

**Affiliations:** Center for Energy Development and Environmental Protection Strategy Research, Jiangsu University, Zhenjiang, Jiangsu, China; Shandong University of Science and Technology, CHINA

## Abstract

With green development becoming a global movement, environmental tax has been adopted by many governments to promote green development. This study analyzes the impact of environmental tax on green development by using a four-dimension dynamical system. The establishment of the system is based on the complex and dynamic interactions among economic development, pollution emissions, resources consumption, and environmental tax, where roles of environmental tax are reflected by the linear parameters. A theoretic analysis shows the complexity of the behavior of the system. Mainly, the existence of chaos is inferred by Lyapunov exponent spectrum and bifurcation diagram, then verified by the presence of a chaotic attractor. An empirical study of the green development dynamical system in China demonstrates the particular evolution paths of economic growth, pollution intensity, and resource intensity under different environmental tax parameters. Results indicate a robust beneficial role of environmental tax on green development. Furthermore, when an environmental tax is imposed, a firm government control, an active consumer awareness, an advanced technology level can stimulate economic growth, decrease pollution intensity, and control the resource intensity. But the government control has a stronger effect. This study provides a viable and promising approach to analyze the role of imposing an environmental tax on green development and may have potential application in other areas and countries.

## Introduction

Green development has become the new contents of sustainable development after the Rio+20 Conference [1]. The Organization for Economic Cooperation and Development defines green development as a solution to promote economic development while prevent the environmental degradation, the loss of biodiversity, and the waste of natural resources [2]. The United Nations Environment Programme (UNEP) regards green development as the process to improve human well-being while significantly reducing environmental risks and mitigating ecological scarcities [3]. The World Bank emphasizes green development as an environmental-friendly and social-inclusive way of economic growth, aiming at efficient use of natural resources and minimizing pollution emission as well as reducing impacts on environment [4]. These universally accepted definitions show that the core of green development is to improve resource utilization and reduce pollution emissions without slowing economic growth [5].

The international society has proposed numerous deals and programs to promote green development. The UNEP proposed the Global Green New Deal which focuses on investing renewable resources, building environmentally friendly society, and increasing energy efficiency [6]. Many countries, such as the US and the UK, have formulated green policies centered on new energy development or low-carbon economic growth [7]. Green development was proposed as China’s national strategy in 2015 [8] to address challenges of resource and environment problems in it. The fast economic growth of China has been accomplished with large consumption, huge pollution, and high emissions. China has almost doubled energy intensity of the developed counties [9]. Haze pollution has attracted international concerns [10] as 70% of the world’s most polluted cities are in China [11]. Nearly half of water sources in China’s key cities are not qualified for drinking [12]. Facing these challenges, the Chinese government adopted lots of measures for sustainable economic growth in the Five-Year Plans. These measures include the promotion of technological innovation, market-oriented reform, industrial structure adjustment, and regional balance development. China’s efforts have brought green effect in the form of the win-win development of resources, environment, and economy [13].

Environmental tax is one of environmental policy measures to fulfill the goals of green development. Various forms taxes have been adopted by many countries. France first imposed forest tax in 1969, which is a kind of resource tax on extraction of raw materials (except for oil and gas) [14]. Very the next year, the US adopted the SO_2_ tax [15]. The tax base was expanded in other countries from SO_2_ to other emission to air (except CO_2_) and water such as CO, NO_x_, taxable water pollutants, and taxable solid pollutants [16, 17]. Japan’s aviation fuel tax levied in 1972 [18] is an example of energy tax, which covers tax on energy products for transport and for stationary use. The CO_2_ tax [19] is also included in this group for statistical reasons. The EU introduced the environmental tax reform (ETR) in the 1990s to shift the burden of taxation away from factors of production to the users of natural resources and pollution [20].

The motivation of this study lies in three aspects. First of all, the fast expansion of green practices urged the authors to study the role of environmental tax in green development from an integrated point of view. Green development means to improve resource utilization and reduce pollution emissions without slowing economic growth. Therefore, the effect of environmental tax on economy, resource, and environment should be considered simultaneously. Secondly, there is a need both in the practical and academic fields to quantify the impact of environmental tax on green development. Most studies are still based on qualitative methods, while quantitative researches are relatively rare. To measure green development, this study divides green development into three qualitative indicators: economic growth, pollution intensity, and resource intensity. Last but not least, it is crucial to study the status of green development in medium and long time periods. Green development is a long-term national strategy, and environmental tax is an economic means to achieve green development. Therefore, the evolution regulation of green development should be analyzed, whether in short, medium, or long terms.

This study aims to analyze the impact of environmental tax on green development. We focus on the evolution of economic growth, pollution intensity, and resource intensity under different environmental tax parameters. For scenario analysis, we acquire the actual green development system based on the underlying data through genetic algorithm. The mathematical results are obtained via a series of comparative analysis on the evolution regulation.

The outlines of this paper are organized as follows. We start with the literature review. In the next section, the GDDS is set up and analyzed. Then we present the dynamic analysis of the GDDS. After that a case study in China is showed, continued with the scenario analysis based on China’s statistical data. In the last section, this paper draws the conclusions.

## Literature review

The existing literature proves that green development is influenced by many factors such as industrial technological, carbon intensity, macroeconomic uncertainty, and environmental regulation. The regional green development performance shows strong spatial dependence and spatial differentiation under the constraints of smog [21]. The green development level in mineral resource-based cities presents a polarizing trend mainly owning to the long-term economic and structural contradictions [22]. Jin et al. [23] show that macroeconomic uncertainty has a more significant negative on urban green development performance in less developed cities than that in developed and coastal cities. Taking Shanghai as an example, Shao et al. [24] find that the performance of industrial green development in Shanghai mainly depends on the technical efficiency change. However, the industrial green development model needs to be further improved due to the industrial technological change is still not stable. The industrial green development performance presents a circuitous downward trend after a transient rise in China. Yang et al. [25] show that the carbon intensity constraint policy has a negative effect on the industrial green production performance. Fu and Geng [26] think the improvement of corporate regulatory compliance can promote green development. Meanwhile, public participation plays an essential role in achieving green development.

Some scholars confirm that imposing environmental tax causes a series of influences on green development from economy, environment, and resources. Researches show different effects of environmental tax on economic growth. Green tax reforms are found to growth improving in a rising economy [27]. Such improvement in economic growth comes from environmental production externality and a tax-shifting towards profits [28]. Furthermore, higher energy tax would lead to higher economic growth if the labor input is mobile and the elasticity of substitution in manufacturing between the scarce factors and energy is less than unity [29]. However, the environmental tax brings a negative effect to economic growth when the impact of environmental tax on socioeconomic and environment are considered comprehensively via a Compute General Equilibrium model [15].

At the same time, a great deal of researchers believe that levying environmental tax is environmentally beneficial in restraining pollution and reducing carbon emissions. Liu et al. [30] demonstrate that environmental tax could foster not only the effects of pollution control but also lessen the losses of ecological environment. Piciu and Tric [31] prove that environmental tax can be returned to polluters, that is to say, environmental tax can reduce pollution and protect the environment under certain conditions. Tamura et al. [32] think environmental tax is effective for total emission control of carbon dioxide only when high eliminate technology with low cost has been developed. Niu et al. [33] find that the environmental tax shocks can drive the reduction of carbon emissions. Wang et al. [34] demonstrate that higher environmental and carbon tax levels in China would achieve more reduction in CO_2_ and air pollutant emission.

From the resource aspect, imposing environmental tax can also save resources to avoid resources waste. Patrik [35] indicates environmental tax is a factor that affects resources recovery although the countries with higher environmental tax, the higher the recovery rate of resources. Piciu and Tric [36] think that environmental tax could encourage the natural resources recycling and it has a significant part of environmental policy in Europe. Amundsen and Schb [37] find levying environmental tax is favorable for countries with depleted resources.

The above analysis of environmental tax on green development mainly comes from one aspect of economic, pollution, and resource. In addition, some studies begin to analyze the impact of environmental tax on green development from two aspects. Based on an endogenous growth model, Bovenberg and Mooij [28] declare ETR could improve environmental quality in one side and boost economic welfare in other side. Ekins et al. [38] adopt econometric modeling to research the effect of ETR in Europe. Their results show that ETR could not only result in long-term economic development but also promote environment benefits. Bosquet [39] demonstrates that environmental tax can reduce carbon emissions significantly and improve the quality of economic environment under certain conditions through a large number of modeling simulations.

The existing research gap is the present literature fail to reflect the impact of environmental tax on the green development from a dynamic and multiple interacting point of view. In fact, the above mentioned researches shed lights on the impact of environmental tax on just one or two laterals of the green development. In previous models, environmental tax is usually taken as an explanatory variables, together with other independent variables in some cases, to explain its impact on economic growth or pollution intensity or resource intensity, such as endogenous growth model [27], energy-environment-economy dynamic stochastic general equilibrium model [33], deterministic optimization model [34], and random coefficient logit model [40]. However, these methods could not analyze the complex interactions among all variables and influencing factors. As a result, the role of environmental tax on green development has not been studied in an integrated view of the economy, environment, and resource.

To fill this gap, this study takes economic growth, pollution emission, resources consumed, and environmental tax as state variables, a nonlinear dynamical system approach can quantitatively analyze the impact of environmental tax on every aspect of green development. A nonlinear dynamical system describes the evolution of a state variable with time in the form of a set of differential equations or discrete mappings. It can analyze the interactions among variables simultaneously by combining multiple variables. There are many applications for nonlinear dynamical systems such as computational fluid dynamics [41], energy-saving and emission-reduction model [42], and resource-economy-pollution system [43].

Compared with the existing literatures, the contribution of this paper is mainly reflected in three folds. (1) Environmental tax is regarded as an equivalent state in a green development dynamical system (GDDS), no longer a control variable as in the existing literature. It seems nature to take environmental tax as a control variable because environmental tax has impact on each aspect of the green development. However, introducing environmental tax as a state variable is of practice base considering that environmental tax is also influenced by different levels of economy, environment, and resource. (2) Green development is analyzed in an integrated manner from economy, environment, and resource sides by using the nonlinear dynamical system approach. This study introduces the continuous-time variables to explore the characteristics of the evolution of every variable with time so that these variables can be considered together. (3) The GDDS is a quantitative model of green development and could provide more possible results. Specifically, this paper uses economic growth, pollution intensity, and resource intensity represent the indicators of economic, pollution, and resource respectively. Therefore, this study can analyze the impact of an environmental tax on green development based on the evolution of three indicators quantitatively.

## The green development dynamical system (GDDS)

This paper establishes a nonlinear dynamical system for green development based on the interactions among resources consumed, economic growth, pollution emission, and environmental tax. It is an extension of the Resource-Economy-Pollution (REP) system in that the environmental tax is added as a state variable.

**Fig 1:**
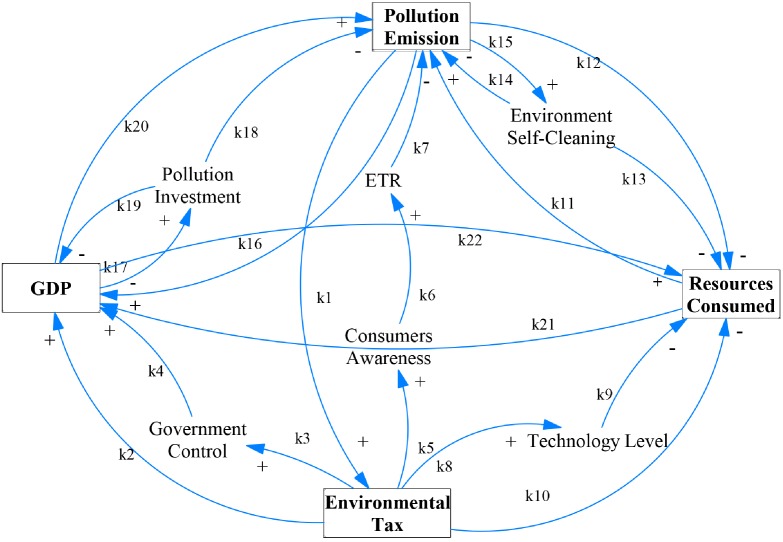
The interactions among the variables of GDDS.

Green development dynamical system (GDDS) is a complicated system including many factors, such as government control, technology level, consumers awareness, pollution investment, ETR, and environment self-cleaning. These factors are closely connected and mutually interacted. Fig 1 qualitatively shows the direction of interactions, where square boxes stand for variables of green development system, unframed blocks indicate influencing factors, the curved arrow labeled by *k*_*i*_ (*i* = 1, 2, ⋯, 22) represents the conductive relationship between certain variables and factors, and the symbol “+” indicates a promotion effect while “-” an inhibition effect between different variables and factors.

We will establish the GDDS according to the complex interactions among resources consumed, economic growth, pollution emission, and environmental tax. Assume that the resources consumed are water resources as well as fossil fuels, such as coal and oil, while low-carbon energy is temporarily ignored [44]. The pollution emission is supposed to be the sum of the discharge of sewage, waste gas, and solid waste. The economy is expected to maintain steady growth for a certain period [45]. Let *x*(*t*), *y*(*t*), *z*(*t*), and *w*(*t*) respectively represent the total resources consumed, the GDP, the amount of pollution, and the amount of environmental tax in an economy during a period. The derivative of these variables is surely some function of the variables themselves. We will find these functions in explicit forms one by one.

Firstly, we analyze the form of $\frac{dw}{dt}=F\left(x,y,z,w\right)$. The main goal of implementation of environmental tax is to reduce air pollution emissions and environment protection. Therefore, *F*(*x*, *y*, *z*, *w*) only contains two variables, namely, pollution *z* and environmental tax *w*. The basic assumption is for environmental tax to grow exponentially, that is, $\frac{dw}{dt}=dw$. When there is severe pollution, environmental tax will be strengthened in a more fierce frequent to raising taxpayers’ awareness of environment protection. Otherwise, when pollution is under control, a loosen environmental tax plan would be carried out [15]. Mathematically, $\frac{dw}{dt}$ is positive when *z* > *N*, but negative when *z* < *N*. As shown by the close path *k*_5_, *k*_6_, *k*_7_, and *k*_1_ in Fig 1, these influences on $\frac{dw}{dt}$ take the form of a product. Then we have

$$\begin{array}{c} \frac{dw}{dt}=dw\left(z-N\right), \end{array}$$ (1)

where *N* is the threshold of pollution discharge to environmental tax [46], the coefficient *d* is the positive function of *k*_1_, *k*_5_, *k*_6_, and *k*_7_.

Secondly, we consider $\frac{dx}{dt}=G\left(x,y,z,w\right)$. It is easy to see that the increase of the total resources consumed is equal to the increase in resources consumption rate, so the resources consumed is positively proportional to its rate, i.e., $\frac{\partial G}{\partial x}>0$, where the partial derivative $\frac{\partial G}{\partial x}$ represents the resources coefficient of the resources consumption rate. From Fig 1, we can find that $\frac{\partial G}{\partial y}$ is positive for an accelerating economy [23]. Because part of waste can be recycled and some pollution can also be turned into resources (see *k*_14_ and *k*_15_), the rate will slow down under the combined effect of pollution conversion and economic growth (see *k*_12_, *k*_13_, and *k*_20_). Then $\frac{dx}{dt}$ gets the third term −*a*_3_*yz*. Imposing environmental tax can reduce the resources consumption through the improvement of the technology level [47] (see *k*_8_, *k*_9_, and *k*_10_), that is, $\frac{\partial G}{\partial w}>0$. The relationship between some variables and factors can be presented by linear terms in the GDDS [48] as linear relationship is the most simple while important approximation of a common phenomenon in the complex real world. Combining the above analysis, we have

$$\begin{array}{c} \frac{dx}{dt}=a_{1}x+a_{2}y-a_{3}yz-a_{4}w. \end{array}$$ (2)

Thirdly, we consider $\frac{dy}{dt}=H\left(x,y,z,w\right)$. Intuitively, the rate related to resources consumed presents a Logistic growth due to the rate is associated with resources consumed *x*(*t*) and the potential share of resources (1 − *x*/*M*) simultaneously. The proportion $\frac{dy}{dt}$ is positive when the level of resources consumption is low, i.e., *x* < *M*, but negative when there is a high resources consumption, i.e., *x* > *M*. In time of resources scarcity and environmental degradation, investment in resource exploring and environmental techniques would be encouraged to alleviate the situation, which might obstruct the development of economy to a certain extent [8] (see *k*_17_ and *k*_19_), i.e., $\frac{\partial H}{\partial y}<0$. The severer pollution, the slower the economy develops, so the pollution variable is negatively proportional to the rate of GDP (see *k*_16_), i.e., $\frac{\partial H}{\partial z}<0$. The rate becomes greater when the amount of environmental tax increases via the government strengthens policy regulations over polluting enterprises (see *k*_2_, *k*_3_, and *k*_4_). Thus, the environmental tax variable is positively proportional to the rate, i.e., $\frac{\partial H}{\partial w}>0$. In summary, we have

$$\begin{array}{c} \frac{dy}{dt}=b_{1}x\left(1-x/M\right)-b_{2}y-b_{3}z+b_{4}w, \end{array}$$ (3)

where *M* is the inflexion of resources consumed to economic growth.

Finally, we consider $\frac{dz}{dt}=I\left(x,y,z,w\right)$. Optimizing the economic impact of environmental issues is an important step in the integrated decision-making process of the development of environment and economy [49]. At present, the pollution will increase with economic growth (see *k*_20_) and large consumption of resources (see *k*_11_). Thus we get the term *c*_1_*xy*. Due to the self-cleaning functions of the environment, such as pollution filtering, waste sink, and waste decomposition, the pollution can slow down (*k*_13_, *k*_14_, and *k*_15_), i.e., $\frac{\partial I}{\partial z}<0$. After charged with environmental tax, tools and techniques are likely to be adopted to decrease the pollution because that consumers have a positive understanding of environmental tax (see *k*_5_, *k*_6_, and *k*_7_), i.e., $\frac{\partial I}{\partial w}<0$ [19]. Then we get

$$\begin{array}{c} \frac{dz}{dt}=c_{1}xy-c_{2}z-c_{3}w. \end{array}$$ (4)

In conclusion, simultaneous Eqs (1)–(4), the green development dynamical system is set up as follows:

$$\begin{array}{c} \left\{ \begin{array}{c} \begin{array}{cc} \frac{dx}{dt}=a_{1}x+a_{2}y-a_{3}yz-a_{4}w, & \\ \frac{dy}{dt}=b_{1}x\left(1-x/M\right)-b_{2}y-b_{3}z+b_{4}w, & \\ \frac{dz}{dt}=c_{1}xy-c_{2}z-c_{3}w, & \\ \frac{dw}{dt}=dw\left(z-N\right), & \end{array} \end{array}\right. \end{array}$$ (5)

where the coefficients *a*_*i*_, *b*_*i*_, *c*_*j*_ (*i* = 1, 2, 3, 4; *j* = 1, 2, 3) together with *M*, *N* are positive.

The indicators of green development can be derived for the variables of the GDDS. Thus, this paper selects economic growth, pollution intensity, and resource intensity as the indicators of green development.

Pollution intensity is devoted to measuring the level of environmental performance. Pollution intensity is the ratio of the total amount of pollution to economic output during a given period [50]. A country’s pollution intensity is usually described as pollutant emissions per GDP, i.e.,

$$\begin{array}{c} \text{pollution}\;\text{intensity}=\frac{\text{pollution}\;\text{discharge}\;\text{during}\;\text{a}\;\text{given}\;\text{period}}{\text{gross}\;\text{domestic}\;\text{product}\;\text{during}\;\text{a}\;\text{given}\;\text{period}}. \end{array}$$

The smaller the pollution intensity, the better the green development performance. According to the dynamical system (5), we can deduce the pollution discharge at time *t* during a given period is *y*(*t*) = *ϕ*_1_(*x*, *k*_1_*y*, *z*, *w*, *t*), and the GDP *z*(*t*) = *ϕ*_2_(*x*, *k*_1_*y*, *z*, *w*, *t*). Thus, the time dependent pollution intensity during a given period can be described as

$$\begin{array}{c} P\left(t\right)=\phi_{1}\left(x,k_{1}y,z,w,t\right)/\phi_{2}\left(x,k_{1}y,z,w,t\right), \end{array}$$ (6)

where *k*_1_ is the pollution discharge coefficient.

Resource intensity is used to measure the resource utilization. Resource intensity is the ratio of the resources consumption to economic output at time *t* during a given period [51, 52]. A country’s resource intensity is usually described as resources consumed per GDP, i.e.,

$$\begin{array}{c} \text{resource}\;\text{intensity}=\frac{\text{resources}\;\text{consumed}\;\text{during}\;\text{a}\;\text{given}\;\text{period}}{\text{gross}\;\text{domestic}\;\text{product}\;\text{during}\;\text{a}\;\text{given}\;\text{period}}. \end{array}$$

A higher resource intensity indicates a higher environmental cost. Let the resources consumed at time *t* during a given period be *x*(*t*) = *φ*_1_(*k*_2_*x*, *y*, *z*, *w*, *t*), and the GDP *z*(*t*) = *φ*_2_(*k*_2_*x*, *y*, *z*, *w*, *t*). Thus, the resource intensity during a given period can be described as

$$\begin{array}{c} R\left(t\right)=\varphi_{1}\left(k_{2}x,y,z,w,t\right)/\varphi_{2}\left(k_{2}x,y,z,w,t\right), \end{array}$$ (7)

where *k*_2_ is the resources consumed coefficient.

## Dynamic analysis of GDDS

The dynamic analysis of the GDDS confirmed by Eq (5) are analyzed in this part, including equilibrium points analysis and complex behaviors of the system.

### Equilibrium points analysis

For an ordinary differential equations, an equilibrium point is a solution that does not change with time. In other words, the equilibrium point is the solution that when the right end of the all equations are zero. For example, the equilibrium point of the system (5) are found by calculating the following nonlinear algebraic equations

$$\begin{array}{c} \left\{ \begin{array}{c} \begin{array}{cc} a_{1}x+a_{2}y-a_{3}yz-a_{4}w=0, & \\ b_{1}x\left(1-x/M\right)-b_{2}y-b_{3}z+b_{4}w=0, & \\ c_{1}xy-c_{2}z-c_{3}w=0, & \\ dw(z-N)=0. & \end{array} \end{array}\right. \end{array}$$ (8)

The number of equilibrium points depends on the values of parameters. However, the origin *E*(0, 0, 0, 0) is always an equilibrium point. This paper selects a possible set of parameters *P*_0_ such that the GDDS would be chaotic. The selection is based on the sign of the eigenvalues of the Jacobian matrix and the divergence of the system.

The Jacobian matrix of Eq (5) at point *E*(*x*, *y*, *z*, *w*) is

$$\begin{array}{c} J=(\begin{array}{cccc} a_{1} & a_{2}-a_{3}z & -a_{3}y & -a_{4}\\ b_{1}-2b_{1}x/M & -b_{2} & -b_{3} & b_{4}\\ c_{1}y & c_{1}x & -c_{2} & -c_{3}\\ 0 & 0 & dw & d(z-N) \end{array}). \end{array}$$ (9)

The characteristic polynomial is

$$\begin{array}{c} f\left(\lambda \right)=\left|J-I\lambda \right|=\lambda \left(\lambda^{3}+p_{1}\lambda^{2}+p_{2}\lambda^{2}+p_{3}\right), \end{array}$$ (10)

where *I* is the fourth-order unit matrix,

$$\begin{array}{c} p_{1}=-a_{1}+b_{2}+c_{2},\\ p_{2}=b_{2}c_{2}-a_{1}c_{2}-a_{1}b_{2}-a_{2}b_{1}+b_{3}c_{1}x+a_{3}b_{1}z+a_{3}c_{1}y^{2}+2\left(a_{1}b_{1}-a_{3}b_{1}xz\right)/M,\\ p_{3}=-a_{1}b_{2}c_{2}-a_{2}b_{1}c_{2}+a_{3}b_{1}c_{2}-a_{1}b_{3}c_{1}x+a_{3}b_{1}c_{1}xy+a_{2}b_{3}c_{1}y-a_{3}b_{3}c_{1}yz+a_{3}b_{2}c_{1}y^{2}+2\left(a_{2}b_{1}c_{2}x-a_{3}b_{1}c_{2}xz-a_{3}b_{1}c_{1}x^{2}y\right)/M. \end{array}$$

This study sees that the equilibrium point is stable if each *p*_*i*_ (*i* = 1, 2, 3) is favorable by the Routh-Hurwitz criterion. For some certain parameters, Eq (10) has unstable saddle-focus points.

The divergence of the GDDS is

$$\begin{array}{c} \nabla V=\frac{\partial \dot{x}}{\partial x}+\frac{\partial \dot{y}}{\partial y}+\frac{\partial \dot{z}}{\partial z}+\frac{\partial \dot{w}}{\partial w}=a_{1}-b_{2}-c_{2}+d\left(z-N\right). \end{array}$$ (11)

Chaos might appear for negative divergence.

Considering further the stable conditions, this study finds a possible parameter set *P*_0_ leading to chaos as shown in Eq (12). On such parameters, this paper has the equilibrium points and their corresponding eigenvalues as shown in Table 1. Analogy to the cases in the 2D and 3D system, this study sees *E*_2_ − *E*_6_ are unstable saddle-focus points, which might lead to chaos.

$$\begin{array}{c} \begin{array}{c} a_{1}=0.065,a_{2}=0.035,a_{3}=0.065,a_{4}=0.026,\\ b_{1}=0.6,b_{2}=0.088,b_{3}=0.07,b_{4}=0.066,\\ c_{1}=0.468,c_{2}=0.065,c_{3}=0.816,d=0.035,\\ M=6.6,N=0.45. \end{array} \end{array}$$ (12)

**Table 1:** Equilibrium points and their corresponding eigenvalues of Eq (5).

Equilibrium point	λ_1_	λ_2_	λ_3_	λ_4_
***E*_1_(0, 0, 0, 0)**	0.1524	-0.1754	-0.0600	-0.2310
***E*_2_(0.0104, −0.274, 0.4500, −0.0347)**	0.0725	-0.2210	−0.0749 + 0.0070*i*	−0.0749 − 0.0070*i*
***E*_3_(0.9606, 7.3824, 0.4500, 4.0342)**	0.1553	-0.2130	−0.1203 + 1.3543*i*	−0.1203 − 1.3543*i*
***E*_4_(8.1926, 4.5817, 0.4500, 21.4948)**	2.7109	-0.2733	−1.3679 + 2.6824*i*	−1.3679 − 2.6824*i*
***E*_5_(0.2157, 0.5522, 0.9292, 0)**	0.0214	-0.1985	−0.0522 + 0.1102*i*	−0.0522 − 0.1102*i*
***E*_6_(4.2367, 0.3663, 12.1048, 0)**	2.8327	-2.7884	-0.1273	0.1927
***E*_7_(8.8345, −0.3542, −24.4057, 0)**	-0.1168	-1.0852	0.0169 + 6.1251*i*	0.0169 − 6.1251*i*
***E*_8_(−0.0870, −0.9314, 0.6318, 0)**	-0.1431	-0.2089	0.0301 + 0.1788*i*	0.0301 − 0.1788*i*

### Complex behaviors

This subsection shows different dynamic behaviors, such as chaotic attractor, limit cycle, and equilibrium, of the GDDS by numerical simulation.

Chaos is a seemingly irregular and complex phenomenon that exists in the real world. Therefore, the emergence of chaos may be an important condition to verify whether the model is consistent with reality [53]. The Lyapunov exponent spectrum and bifurcation diagram are used to prove the appearance of chaos. Since the main goal of environmental tax is to reduce pollution, we select the bifurcation parameter to be *c*_2_ which is directly related to pollution. All results are based on the initial value [0.196, 0.36, 0.88, 0.29] and parameters in set *P*_0_ while *c*_2_ varying in the interval [0.05 0.13].

**Fig 2:**
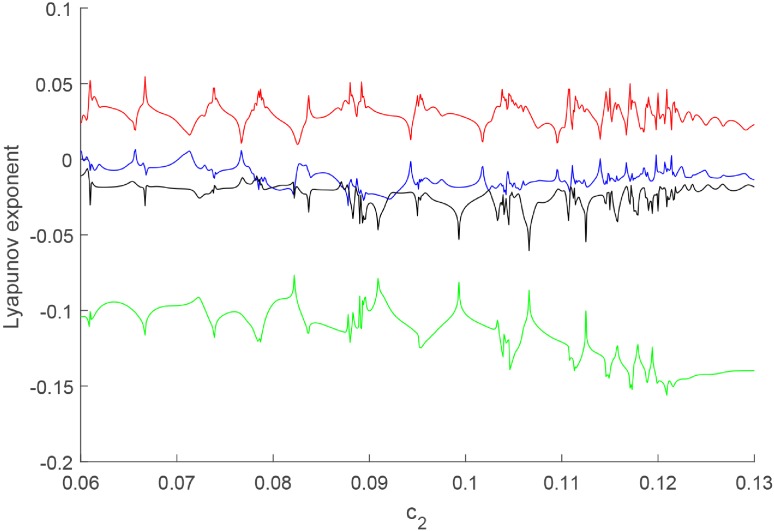
Lyapunov exponent spectrum.

Firstly, the existence of chaos is verified by the Lyapunov exponent spectrum. The Lyapunov exponent spectrum is shown in Fig 2. The system might be chaotic at certain parameters where the maximum Lyapunov exponent is positive and the sum of all Lyapunov exponents is negative.

**Fig 3:**
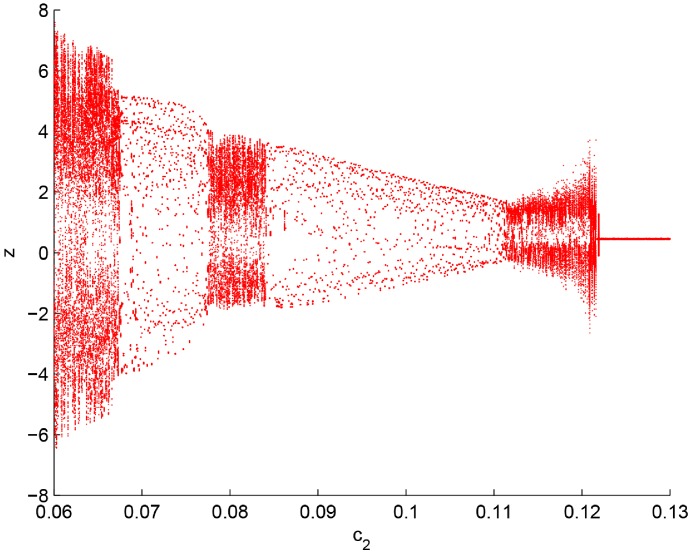
Bifurcation diagram of *z* with parameter *c*_2_.

Secondly, the existence of chaos is further tested by bifurcation diagram. The bifurcation diagram is as shown in Fig 3. An abrupt bifurcation occurs in the system about *c*_2_ = 0.122, that is to say, *c*_2_ = 0.122 is the critical value between a stable and unstable state of the GDDS. Chaos would appear at parameters smaller than the critical value.

Among the four state variables, the pollution variable *z* has a more close relation with environmental tax because the base of environmental tax is a physical unit that has a proven, specific, and negative impact on the environment. Therefore, we only select the bifurcation diagram of the pollution variable *z*. Furthermore, *z* is not isolated. Actually, the four state variables *x*, *y*, *z*, and *w* in the GDDS are interrelated. Once chaos is inferred from the bifurcation diagram of *z*, the same inference can also be drawn from bifurcation diagrams of other variables.

**Fig 4:**
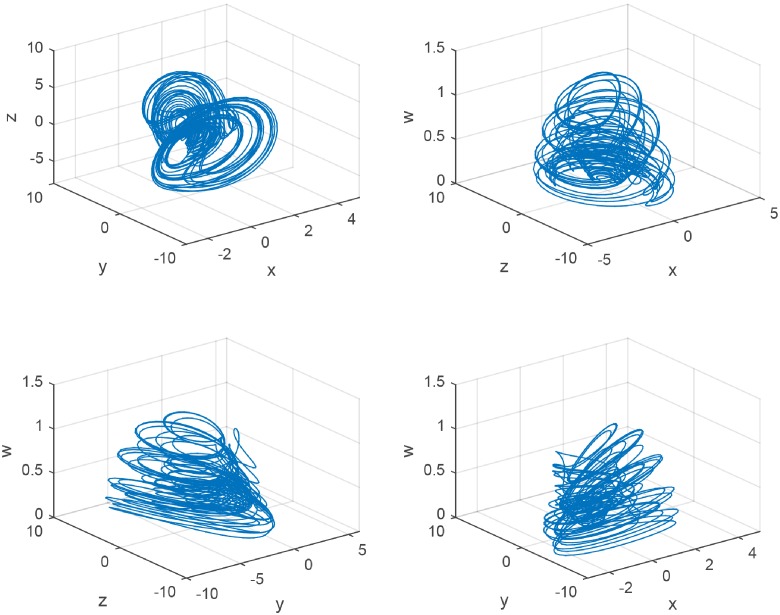
The 3D view of the GDDS chaotic attractor with *c*_2_ = 0.065.

Finally, a chaotic attractor occurs as shown in Fig 4 when *c*_2_ = 0.065. The chaotic attractor of GDDS is a new attractor compared with the previous chaotic attractors such as Lorenz attractor, Chen attractor, and Lü attractor. It has different chaotic behaviors, different equilibrium points, and various types of equilibrium points based on above analysis.

**Fig 5:**
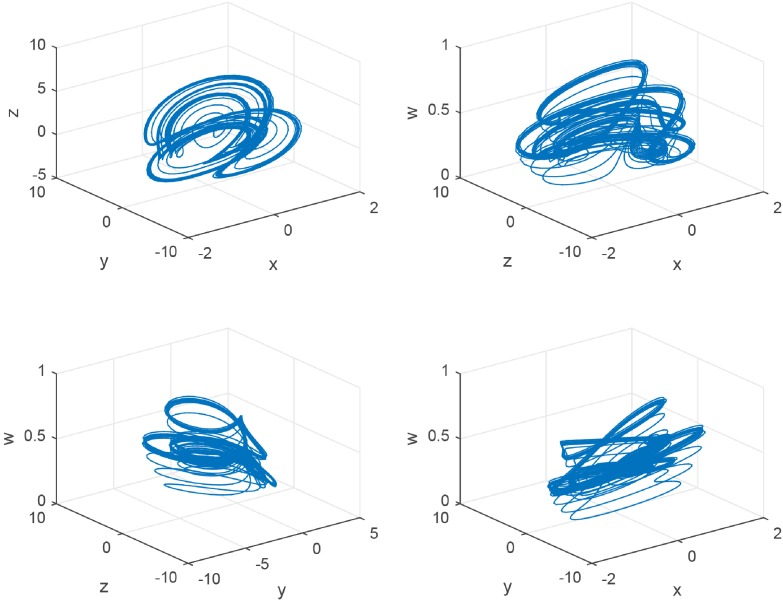
The 3D view of the GDDS chaotic attractor with *c*_2_ = 0.071.

Generally, there are different chaotic attractors when these parameters changed. For system (5), in addition to the chaotic attractor as shown in Fig 4, we will have an another new chaotic attractor (Fig 5) when *c*_2_ changes into 0.071.

**Fig 6:**
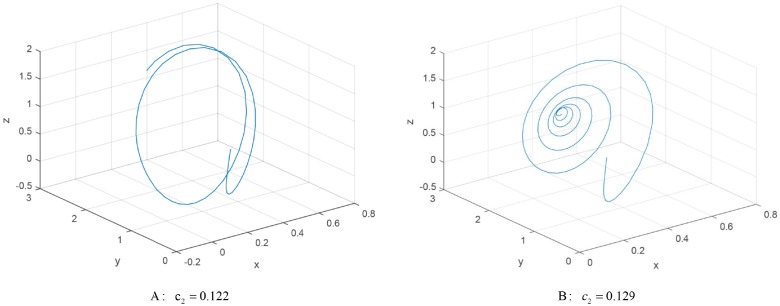
The stable state of the GDDS. **A: The limit circle with** *c*_2_ = 0.122. **B: The equilibrium with** *c*_2_ = 0.129.

The limit cycle of the GDDS can be obtained when the maximum Lyapunov exponent is zero. A in Fig 6 indicates a limit circle of the GDDS. We can see that the trajectories of the GDDS present a periodic phenomenon when *c*_2_ = 0.122.

We can get stable equilibriums when the maximum Lyapunov exponent is negative. B in Fig 6 indicates a stable equilibrium of the GDDS with *c*_2_ = 0.129. It means that the trajectories of the GDDS convergence to the equilibrium at the same time.

The above analysis shows that the GDDS has different trajectories under different parameters: a limit cycle, an equilibrium point, or chaos. For an actual system, its evolution has already been determined by its system parameters and the initial conditions. However, its phase may be changed once there are some perturbation to the parameters. Like the theoretic model does, the actual system may change from one of the above mentioned behavior to another under different sets of parameters [46]. A chaotic state is not conducive to the development of economic and social. Different stable states correspond to different socioeconomic development reality. Therefore, we want to find an empirical way to adjust the parameter to grantee a desired evolution results.

## A case study in China

This section exemplifies a stable case of the GDDS. First, this paper obtains the required data from the China Statistical Yearbook and process the data through the analytic hierarchy process. Then, this paper gets a set of actual parameters *P*_1_ by genetic algorithm based on actual data. Finally, the phase diagram of the actual system is obtained.

### Data and preliminary process

In the practice, the variables for resources, economic, pollution, and environmental tax are the combination of several elements. This study takes the resources variable *x*(*t*) as the sum of consumption of coal, oil, natural gas, and water resources. The pollution variable is *z* = 0.5788*z*_1_ + 0.8059*z*_2_ + 0.1247*z*_3_, where *z*_1_, *z*_2_, and *z*_3_ represent the emissions of industrial waste water, industrial exhaust, industrial solid waste production respectively [43]. The environmental tax *w* = 1.4*z*_1_ + 1.14*z*_2_ + 5*z*_3_ with the coefficients in turn being the money paid for per pollution equivalent chemical oxygen demand, per pollution equivalent sulfur dioxide, and per ton of coal waste [54].

The underlying data come from the China Statistical Yearbook (2007-2016) and are processed by *z*–score standardization. This paper then gets the variable values for the GDDS as listed in Table 2.

**Table 2:** Experimental values for *x*(*t*), *y*(*t*), *z*(*t*), and *w*(*t*).

Year	*x*(*t*)	*y*(*t*)	*z*(*t*)	*w*(*t*)	Year	*x*(*t*)	*y*(*t*)	*z*(*t*)	*w*(*t*)
**2007**	-0.2701	-0.2166	0.6828	0.4354	**2012**	0.7740	0.6066	0.9514	1.0188
**2008**	-0.1677	-0.0664	0.4772	0.3080	**2013**	0.9015	0.7738	0.7682	0.8704
**2009**	-0.0279	0.0237	0.3570	0.2166	**2014**	0.9359	0.9223	0.6697	0.7900
**2010**	0.3132	0.2185	0.2841	0.1721	**2015**	0.9544	1.0597	0.5641	0.6951
**2011**	0.5783	0.4510	1.0047	1.0727	**2016**	1.0086	1.2275	-0.7592	-0.5791

### Genetic algorithm and parameters identification

This study uses the genetic algorithm (GA) to identify the actual parameters. The GA is an optimization procedure modeling the natural selection in biological evolution. In a genetic algorithm, a population of candidate solutions to an optimization problem is evolved toward better solutions. Each candidate solution has a set of genotype which can be mutated and altered. A GA proceeds to initialize a population of solutions and then to improve it through repetitive application of the mutation, crossover, inversion, and selection operators. GA has been successfully applied in identifying parameters of actual nonlinear dynamical systems [55].

To using the GA, we rewrite the GDDS as a vector form:

$$\begin{array}{c} \dot{X}\left(t\right)=f\left(X\left(t\right),\alpha \right), \end{array}$$ (13)

where *X* represents the state of the system, *α* is the system parameter. We then discrete it as:

$$\begin{array}{c} X(k+1)=X(k)+f(X(k))=F(X(k),\alpha ). \end{array}$$ (14)

Therefore, the parameter identification of the GDDS is equivalent to the nonlinear optimization problem:

$$\begin{array}{c} min\;\frac{1}{2}\sum_{k=1}^{T}\|X(k+1)-F(X(k),\alpha ){\|}^{2},\;\;\;s.t.\;\alpha_{i}>0. \end{array}$$ (15)

The identification process is carried out on a PC by using the MATLAB software. The population size is set as 200, the crossover rate is 0.4, the mutation rate is 0.1, error tolerance is 10^−6^. This paper then gets the identified parameter set *P*_1_ as shown in Eq (16). This set is distinct from the set *P*_0_. Consequently, different phenomena of other chaos may occur.

$$\begin{array}{c} \begin{array}{c} a_{1}=0.1342,a_{2}=0.5525,a_{3}=0.7955,a_{4}=0.0408,\\ b_{1}=0.0094,b_{2}=0.5517,b_{3}=0.0396,b_{4}=0.2488,\\ c_{1}=0.6705,c_{2}=0.5828,c_{3}=0.1037,d=0.4092,\\ M=0.1951,N=0.6873. \end{array} \end{array}$$ (16)

**Fig 7:**
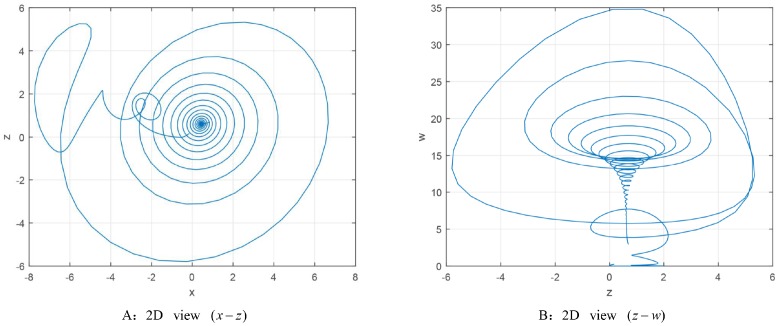
The 2D phase diagram of the 4D actual system.

This paper gets a stable state for the actual system. Fig 7 shows the 2D-phases of the actual system on the parameter set *P*_1_ and the initial values [0.14, −0.25, 0.17, 0.25]. It indicates that the system evolves to a stable point. Furthermore, this point is calculated to be [0.8509, 1.2424, 0.6872, 2.9720] by MATLAB.

## Scenario analysis

This section discusses the impact of environmental tax on economic growth, pollution intensity, and resource intensity. The actual GDDS identified in the above section is used as the benchmark. This paper analyzes the evolution of the three indicators for different values of parameters *a*_4_, *b*_4_, and *c*_3_. These parameters are closely related with environmental tax. We recall that *a*_4_, *b*_4_, and *c*_3_ represent the technology level, the government control, and the consumer awareness respectively.

This paper sets three scenario cases when environmental tax is employed. The benchmark case corresponds to using parameters in set *P*_1_, while the medium case with a greater parameter, and the strong case with the largest parameter. The case without environmental tax is called the null case.

### The impact on economic growth

This paper first analyzes the impact on economic growth of governmental control and consumer awareness.

**Fig 8:**
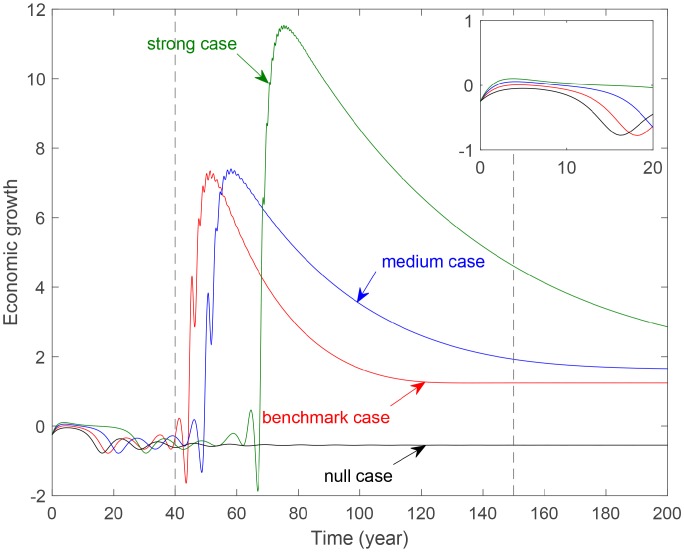
Evolution of economic growth with different government control parameter *b*_4_. **Benchmark case**: *b*_4_ = 0.2488. **Medium case**: *b*_4_ = 0.4488. **Strong case**: *b*_4_ = 0.6488. **The vertical dotted lines separate the evolution period into three time periods. The sub-figure in the upper-right corner is a partially enlarged view. The figures after this have the same meanings as this figure**.

Fig 8 shows the evolution of economic growth with different levels of government control. Firstly, curves for three cases with environmental tax have greater fluctuation than the null case. This indicates that imposing environmental tax has obvious impacts on the economy. Secondly, the evolution period can be divided into three periods: the initial stage, the fluctuation phase, and the stable period. In the initial stage, no significant differences exist for the four curves. In the fluctuation period, the strong case has the highest but delayed peak. This indicates that the stronger the government control, the bigger yet later the economy peak. In the stable period, economic growth decrease monotonously then levels off according to the strength. This indicates that a higher government control could promote economic development in the long run.

**Fig 9:**
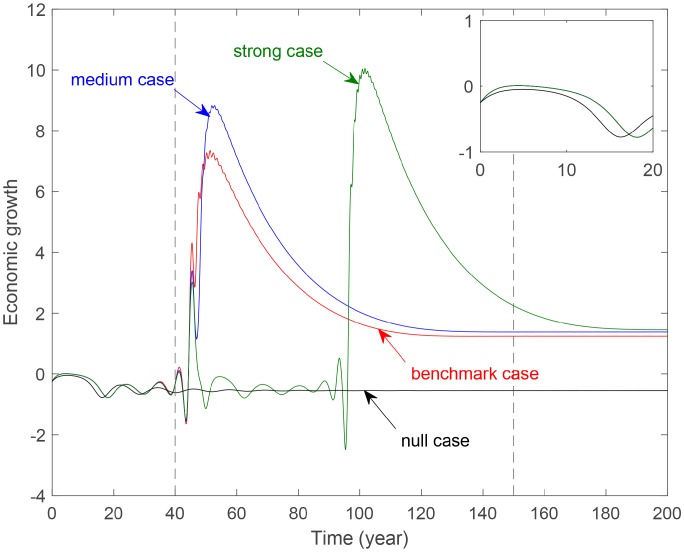
Evolution of economic growth with different consumers control parameter *c*_3_. **Benchmark case**: *c*_3_ = 0.1037. **Medium case**: *c*_3_ = 0.1437. **Strong case**: *c*_3_ = 0.1637.

Fig 9 reveals the evolution of economic growth with different levels of consumer awareness. Curve for the null case is gentler than the three cases with environmental tax and is almost always below these curves. This demonstrates that imposing environmental tax can promote economic growth. In the initial period, these four curves roughly coincide. In the fluctuation period, the greater the consumer awareness, the higher and far more right the peak. In the stable period, all the curves tend to a same approximate stage. But it takes more time for the strong case. This demonstrates the improvement of consumer awareness can also improve economy in the long term. But the effect is weaker than that of the government control.

### The impact on pollution intensity

Then, this paper discusses the impact on pollution intensity of government control.

**Fig 10:**
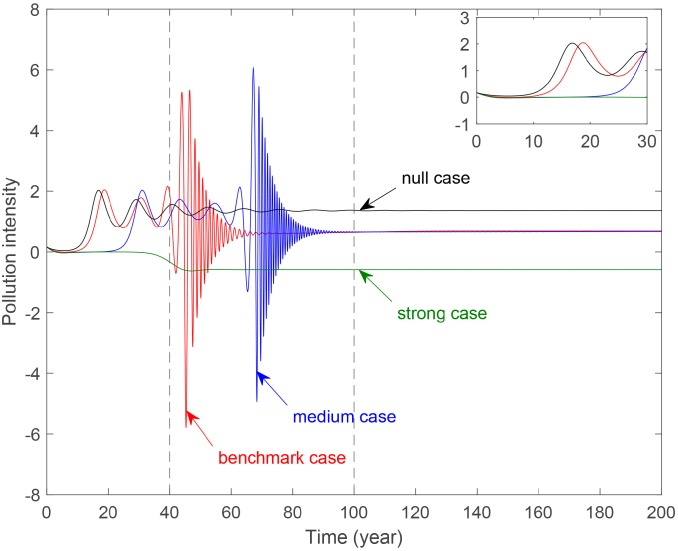
Evolution of pollution intensity with different government control parameter *b*_4_. **Benchmark case**: *b*_4_ = 0.2488. **Medium case**: *b*_4_ = 0.6488. **Strong case**: *b*_4_ = 0.7488.

Fig 10 represents the evolution of pollution intensity with different degrees of government control. Curves for three cases with environmental tax are below that of the null case. This shows that imposing environmental tax plays a significant role in reducing pollution. In the initial period, the strong case curve is below the other three curves. In the fluctuation period, curves for the benchmark case and medium case have strong fluctuation, and the other curves are almost stable. This indicates there is an upper limit of government control. Under the border, the corresponding curves fluctuate without determined modes. But in the stable period, These curves are leveling off between the null case and the strong case. This shows that the stronger government control has significant impacts on reducing pollution.

### The impact on resource intensity

Finally, this study focuses on the impact on resource intensity of government control and technology level.

**Fig 11:**
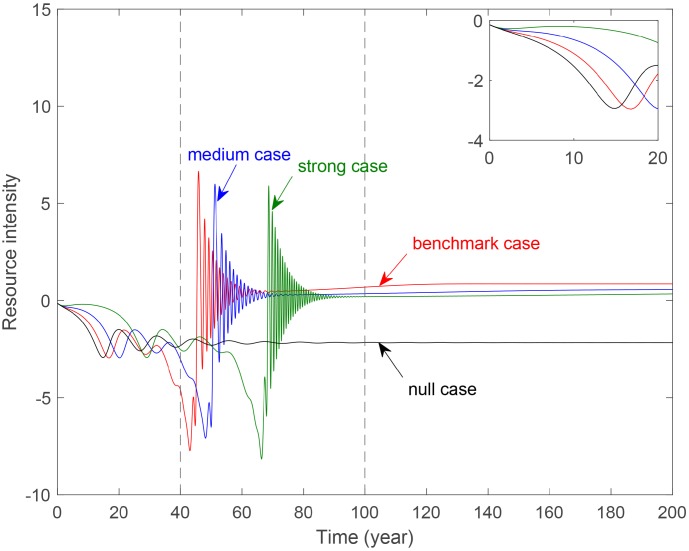
Evolution of resource intensity with different government control parameter *b*_4_. **Benchmark case**: *b*_4_ = 0.2488. **Medium case**: *b*_4_ = 0.4488. **Strong case**: *b*_4_ = 0.6488.

Fig 11 shows the evolution of resource intensity with different strength of government control. Note that the null case curve is almost below than the curves for three cases with environmental tax. This indicates that the increase in resources consumed makes the economy negative so that the resource intensity is negative without the environmental tax, that is, imposing environmental tax could help save resources. In the initial period, all curves are negative and the strong case locates the highest. In the fluctuation period, curves for the three cases with environmental tax fluctuate more frequently while the null case curve is almost stable. The stronger the government control, the later the fluctuations occur. But the peak is not changed much with the increase of government control. In the stable period, the four curves are tend to stable, and the stable value become smaller and smaller as the government control increases. This indicates that the strong government control could manage resources effectively.

**Fig 12:**
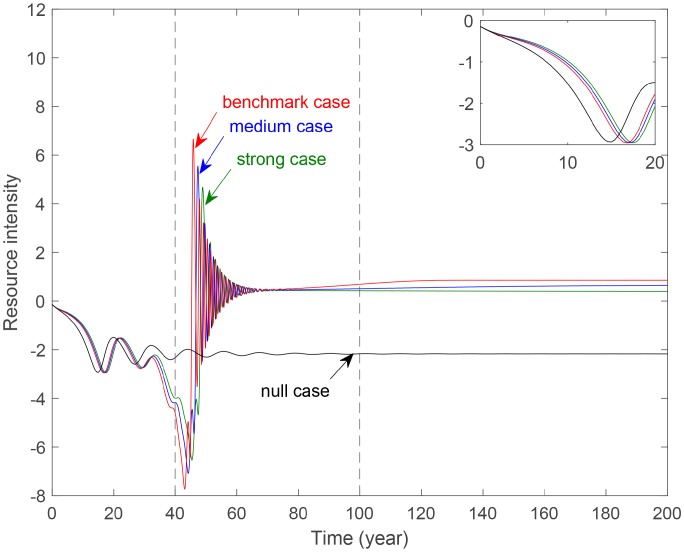
Evolution of resource intensity with different technology level parameter *a*_4_. **Benchmark case**: *a*_4_ = 0.0008. **Medium case**: *a*_4_ = 0.0208. **Strong case**: *a*_4_ = 0.0408.

Fig 12 demonstrates the evolution of resource intensity with different levels of technology. The variation patterns are similar to these in Fig 12. For the length of the paper, this paper omits the detail explanation. This paper summarizes that improve the level of technology can also save resources and avoid resources waste to manage resources.

## Conclusions

The international community has regarded environmental tax as a real policy instrument for green development. This paper shows the evolution of economic growth, pollution intensity, and resource intensity when imposing environmental tax from a perspective of dynamical system. A four-dimension dynamical system is constructed to model the complex interactions among the variables of resources, economy, pollution, and environmental tax. Stability of the GDDS is studied by dissipative analysis and Routh-Hurwitz criteria and this system is proved to be chaotic by the Lyapunov exponent spectrum and bifurcation diagram. By using genetic algorithm, system parameters are identified under which the benchmark case of China is stable. Evolution paths under different scenarios are illustrated to display the role of environmental tax on green development.

This paper confirms the view in the literature mathematically that imposing environmental tax plays an active part in green development. The role of environmental tax on green development is reflected in this study by the comparison of the evolution of green development indicators between different scenarios. On one hand, when an environmental tax has been levied, three indicators all deviate away from their null case without environmental tax. The relative locations of the indicator curves show that environmental tax can promote economic growth, save resources, and reduce pollution. The influence becomes significantly greater in the long run. On the other hand, results of the differences in the evolution paths under selected environmental tax parameters indicate possible ways to promote green development. The ways include reinforcing government control, increasing consumers awareness as well as improving technology level. Among them, the government control surpasses the others. This indicates the necessity to enhance the government’s functions such as further improve China’s government supervision system, establish and improve various environmental tax systems, formulate strict emission standards, and continuously supplement multiple standards. The enhancement might be practical for China as the country has a powerful government system. For other market-oriented economies, although the dynamical system approach can still be used, the preferred consideration is advanced technology and higher consumers awareness rather than the government control.

There are still some limitations in this research. Firstly, results of this research may be vulnerable. As this study applies a small set of data and the status quo in China, different results may be drawn if study area changes or more abundant data are available. Secondly, the linear terms in the theoretical dynamical model are simple assumptions of the relations between green development variables. More sophisticated assumptions and terms may be made and derived. Thirdly, this study focuses on green development in a country or regional level. Green development in industries or sectors has not been involved. This study also provides some possible directions for future researches. One direction can be the application of the GDDS in other countries and regions. The second direction can be the modification of the GDDS. The linear assumption can be weakened to more complex forms such as quadratics to set up new green development models. Future studies can also focus on theoretical modeling or empirical application of GDDS in industries or sectors.
